# Unicondylar knee arthroplasty versus total knee arthroplasty in adults with isolated medial osteoarthritis: A matched study protocol

**DOI:** 10.1097/MD.0000000000021868

**Published:** 2020-08-28

**Authors:** Zifei Yin, Pingkang Qian, Xiaofeng Wu, Feng Gao, Feng Xu

**Affiliations:** Department of joint, Kunshan hospital of Traditional Chinese Medicine affiliated to Nanjing University of Traditional Chinese Medicine, Jiangsu Province, China.

**Keywords:** functional score, isolated medial osteoarthritis, protocol, total knee arthroplasty, unicondylar knee arthroplasty

## Abstract

**Background:**

The choice between unicondylar knee arthroplasty (UKA) and total knee arthroplasty (TKA) is likely to have long-term implications for patient-reported health outcomes. However, high-quality studies that compare the outcomes of TKA and UKA and their effects are still lacking in the literature. Thus, the aim of the present study was to compare the UKA and TKA techniques with regard to functional outcomes and perioperative complications in patients who had isolated medial osteoarthritis.

**Methods:**

This was a retrospective, single-center, matched-controlled study performed with approval of our hospital (Kunshan hospital of Traditional Chinese Medicine affiliated to Nanjing University of Traditional Chinese Medicine), with the ethics number KZY2020–37. To reduce the effect of selection bias and potential confounding in this observational study, a 1:1 matching algorithm was applied. The groups were split by sex, age to within 6 years, and body mass index within 5 kg/m^2^. Thus, we retrospectively reviewed the records of 240 consecutively enrolled patients who underwent UKA and 240 patients who underwent TKA from January 2013 to June 2015 from the database of our institution. Written informed consent was obtained from all subjects participating in the trial. Clinical outcomes included range of motion, Short Form 12 score, new Knee Society Score, Western Ontario and McMaster Universities Arthritis Index, and the complications. The outcome measures were evaluated by a physiotherapist and were assessed preoperatively and postoperatively at 6 months and 2 years. The mean follow-up time was 3 years.

**Conclusion:**

We hypothesized that there was no significant difference between the 2 groups in terms of postoperative outcomes.

**Trial registration:**

Our study was registered in Research Registry (researchregistry5828).

## 1 Introduction

Changes in demographics and physical activities of the younger population have increased the number of patients with medial unicompartmental knee osteoarthritis requiring surgical intervention.^[1–3]^ Conservative surgical procedures such as core decompression, arthroscopic debridement, or high tibial osteotomy are effective in the early stage of the disease, but when bone-on-bone osteoarthritis occurs, a knee arthroplasty is often required.^[4–6]^

Unicondylar knee arthroplasty (UKA) and total knee arthroplasty (TKA) are both accepted management options for patients with end-stage isolated medial compartmental joint disease. UKA was first introduced in the 1970s as an alternative to TKA for single-compartment osteoarthritis.^[7]^ UKA preserves bone tissue that will be valuable if prosthetic revision is needed. In addition, UKA has fewer complications, requires less rehabilitation, and may provide a better range of motion and superior function compared with TKA.^[8–10]^ However, UKA is not universally employed by all surgeons as there is an associated higher revision rate when compared to TKA. The higher revision rates of UKA are thought to be primarily due to component malpositioning, postoperative limb malalignment, and surgeon volume. Therefore, TKA may be preferable to UKA in young patients with severe unicompartmental disease as it offers more predictable outcomes and lower revision rates.^[11–16]^

The choice between UKA and TKA is likely to have long-term implications for patient-reported health outcomes. However, high-quality studies that compare the outcomes of TKA and UKA and their effects are still lacking in the literature. Thus, the aim of the present study was to compare the UKA and TKA techniques with regard to functional outcomes and perioperative complications in patients who had isolated medial osteoarthritis. We hypothesized that there was no significant difference between the 2 groups in terms of postoperative outcomes.

## 2 Materials and methods

### 2.1 Study design

This was a retrospective, single-center, matched-controlled study performed with approval of our hospital (Kunshan hospital of Traditional Chinese Medicine affiliated to Nanjing University of Traditional Chinese Medicine), with the ethics number KZY2020–37. To reduce the effect of selection bias and potential confounding in this observational study, a 1:1 matching algorithm was applied (Table 1). The groups were split by sex, age to within 6 years, and body mass index within 5 kg/m^2^. Thus, we retrospectively reviewed the records of 240 consecutively enrolled patients who underwent UKA and 240 patients who underwent TKA from January 2013 to June 2015 from the database of our institution. Written informed consent was obtained from all subjects participating in the trial. The trial protocol was also registered at the Research Registry (researchregistry5828). All data were collected prospectively.

**Table 1:**
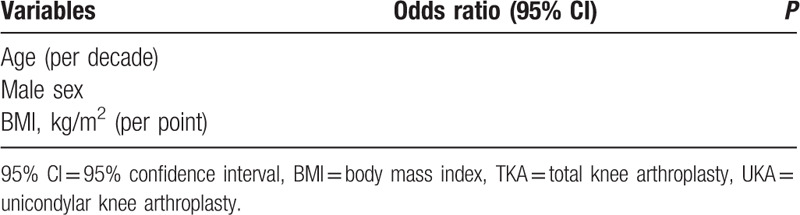
Odds of undergoing TKA compared with UKA based on propensity score model.

### 2.2 Inclusion and exclusion criteria

Inclusion criteria for this study included the following: diagnosis of osteoarthritis or osteonecrosis limited to the medial compartment; passively correctible varus deformity of <10 degree; fixed flexion deformity <15 degree; maximum knee flexion >90 degree; and patients between 18 and 80 years of age. Exclusion criteria included those with ligament insufficiency, inflammatory arthritis, haemochromatosis, chondrocalcinosis, a deformity requiring augmentation, neurological movement disorders, pathology of the feet, ankles, hips, or opposite knee causing significant pain or gait alterations.

### 2.3 Techniques

All of the operations were performed by a single senior surgeon, using a tourniquet and a medial parapatellar approach. Intraoperatively, single-shot cefazolin 2 g (or clindamycin 600 mg in case of incompatibility of penicillin) for infection prophylaxis was given to all patients.

#### 2.3.1 UKA group

The surgeries were performed with a standard minimal invasive midline vertical incision and medial parapatellar approach; the patella was removed laterally but not dislocated or everted. In all cases, the uncemented Oxford Phase 3 (Zimmer Biomet) was used for the UKA procedure. Implants were fixated cementless, hybrid cemented (cemented tibial and cementless femoral component) or fully cemented depending on bone stock and age. Bone resections and implant insertion were performed according to the manufacturers’ manual.

#### 2.3.2 TKA group

The surgeries were performed with a medial parapatellar approach. The Vanguard Complete Total Knee (Zimmer Biomet) with posterior stabilized insert was used for the TKA procedure. Patella resurfacing was performed in all patients. Implants were fixated cementless, hybrid cemented (cemented tibial and cementless femoral component), or fully cemented depending on bone stock and age. Cruciate-retaining implants were used in all cases, as none of the patients were inflammatory arthritis or required posterior cruciate ligament resection.

### 2.4 Postoperative Protocol

Postoperatively, all patients in both cohorts were given short-acting narcotics (typically oxycodone–acetaminophen) and continued on nonsteroidal anti-inflammatory drugs and neurologic agents if not contraindicated. Short-acting intravenous narcotics and antiemetic agents were available before discharge. Patients deemed to be low risk for deep venous thrombosis were prescribed aspirin twice a day postoperatively for venous thromboembolism prophylaxis, whereas patients deemed to be at higher risk were placed on 14 days of low-molecular-weight heparin once daily followed by 1 additional month of twice daily aspirin.

### 2.5 Outcome measures

Clinical outcomes included range of motion, Short Form 12 (SF-12) score, new Knee Society Score (KSS), Western Ontario and McMaster Universities Arthritis Index (WOMAC), and the complications. The outcome measures were evaluated by a physiotherapist and were assessed preoperatively and postoperatively at 6 months and 2 years. The mean follow-up time was 3 years (Table 2).

**Table 2:**
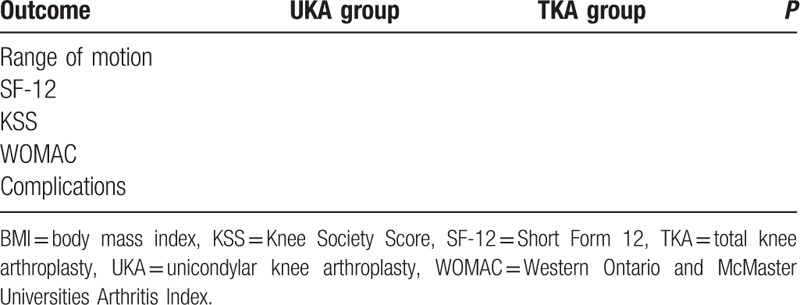
The postoperative outcomes in the 2 groups.

The SF-12 is a 12-item self-assessment health questionnaire that evaluates overall generic physical health and mental health. The physical and mental scores range from 0 (worst) to 100 (best). This score was included to account for confounding variables of generic physical and mental health.

The new KSS is broadly applicable across sex, age, activity level, and implant type. It is a highly responsive outcome measuring tool that may be applied in both the clinical and research settings to elucidate the profound variability of activity levels, function, and satisfaction after knee arthroplasty.

The WOMAC is thought to be the primary measure of efficacy for osteoarthritis trials, and is a self-administered health status measure that assesses the dimensions of pain, stiffness, and function either separately or as an overall index.

### 2.6 Statistical analysis

Statistical analyses were conducted using SPSS v22.0 software (IBM, Chicago, IL). Conformity of the data to normal distribution was tested with the Kolmogorove-Smirnov test. Independent 2-samples *t* test was used for comparison of continuous variables and Pearson *χ*^2^ test was used for comparison of categorical variables. Results were evaluated in a confidence interval of 95% and at a significance level of *P* < .05.

## 3 Discussion

TKA remains the most reliable procedure for relieving patient pain and improving function associated with end-stage degenerative joint disease of the knee. UKA has been demonstrated to be a reliable procedure in appropriately selected patients. The aim of the present study was to compare the UKA and TKA techniques with regard to functional outcomes and perioperative complications in patients who had isolated medial osteoarthritis. We hypothesized that there was no significant difference between the 2 groups in terms of postoperative outcomes.

## Author contributions

Zifei Yin wrote the first draft of the article of the study protocol. Pingkang Qian and Feng Xu are responsible for review and editing. Zifei Yin, Pingkang Qian, and Feng Xu are responsible for managing the project and conducting a formal analysis. Feng Gao and Xiaofeng Wu are responsible for data curation. Feng Xu received the funding to ensure that this study could go forward. All authors have contributed to the design and implementation of the study.
